# Nonsteroidal Anti-inflammatory Drugs Alter the Microbiota and Exacerbate *Clostridium difficile* Colitis while Dysregulating the Inflammatory Response

**DOI:** 10.1128/mBio.02282-18

**Published:** 2019-01-08

**Authors:** Damian Maseda, Joseph P. Zackular, Bruno Trindade, Leslie Kirk, Jennifer Lising Roxas, Lisa M. Rogers, Mary K. Washington, Liping Du, Tatsuki Koyama, V. K. Viswanathan, Gayatri Vedantam, Patrick D. Schloss, Leslie J. Crofford, Eric P. Skaar, David M. Aronoff

**Affiliations:** a Department of Pathology, Microbiology, and Immunology, Vanderbilt University School of Medicine, Nashville, Tennessee, USA; b Department of Medicine, Vanderbilt University School of Medicine, Nashville, Tennessee, USA; c Department of Microbiology and Immunology, University of Michigan, Ann Arbor, Michigan, USA; d School of Animal and Comparative Biomedical Sciences, University of Arizona, Tucson, Arizona, USA; e Center for Quantitative Sciences, Department of Biostatistics, Vanderbilt University School of Medicine, Nashville, Tennessee, USA; University of Oklahoma Health Sciences Center

**Keywords:** *Clostridium difficile*, colitis, gut inflammation, immune dysfunction, immune response, inflammation, intestinal immunity, prostaglandin

## Abstract

Clostridium difficile infection (CDI) is a major public health threat worldwide. The use of nonsteroidal anti-inflammatory drugs (NSAIDs) is associated with enhanced susceptibility to and severity of CDI; however, the mechanisms driving this phenomenon have not been elucidated. NSAIDs alter prostaglandin (PG) metabolism by inhibiting cyclooxygenase (COX) enzymes. Here, we found that treatment with the NSAID indomethacin prior to infection altered the microbiota and dramatically increased mortality and the intestinal pathology associated with CDI in mice. We demonstrated that in C. difficile-infected animals, indomethacin treatment led to PG deregulation, an altered proinflammatory transcriptional and protein profile, and perturbed epithelial cell junctions. These effects were paralleled by increased recruitment of intestinal neutrophils and CD4^+^ cells and also by a perturbation of the gut microbiota. Together, these data implicate NSAIDs in the disruption of protective COX-mediated PG production during CDI, resulting in altered epithelial integrity and associated immune responses.

## Abstract (precis)

Clostridium difficile infection (CDI) is a spore-forming anaerobic bacterium and leading cause of antibiotic-associated colitis. Epidemiological data suggest that use of nonsteroidal anti-inflammatory drugs (NSAIDs) increases the risk for CDI in humans, a potentially important observation given the widespread use of NSAIDs. Prior studies in rodent models of CDI found that NSAID exposure following infection increases the severity of CDI, but mechanisms to explain this are lacking. Here we present new data from a mouse model of antibiotic-associated CDI suggesting that brief NSAID exposure prior to CDI increases the severity of the infectious colitis. These data shed new light on potential mechanisms linking NSAID use to worsened CDI, including drug-induced disturbances to the gut microbiome and colonic epithelial integrity. Studies were limited to a single NSAID (indomethacin), so future studies are needed to assess the generalizability of our findings and to establish a direct link to the human condition.

## INTRODUCTION

Clostridium difficile is the most commonly reported nosocomial pathogen in the United States and an urgent public health threat worldwide (1). C. difficile infection (CDI) manifests as a spectrum of gastrointestinal disorders ranging from mild diarrhea to toxic megacolon and/or death, particularly in older adults (2). The primary risk factor for CDI is antibiotic treatment, which perturbs the resident gut microbiota and abolishes colonization resistance (3). However, factors other than antibiotic exposure increase the risk for CDI and the incidence of cases not associated with the use of antimicrobials has been on the rise (4). Defining mechanisms whereby nonantibiotic factors impact CDI pathogenesis promises to reveal actionable targets for preventing or treating this infection.

Recently, several previously unappreciated immune system, host, microbiota, and dietary factors have emerged as modulators of CDI severity and risk. The food additive trehalose, for example, was recently shown to increase C. difficile virulence in mice, and the widespread adoption of trehalose in food products was implicated in the emergence of hypervirulent strains of C. difficile (5). Similarly, excess dietary zinc had a profound impact on severity of C. difficile disease in mice, and high levels of zinc altered the gut microbiota and increased susceptibility to CDI (6). Importantly, there is a growing body of evidence of the essential role of the innate immune response and inflammation in both protection against and pathology of CDI (7–9). Mounting a proper and robust inflammatory response is critical for successful clearance of C. difficile, and the immune response can be a key predictor of prognosis (3, 10). In this context, specific immune mediators can facilitate both protective and pathogenic responses through the activity of molecules such as interleukin-23 (IL-23) and IL-22, and an excessive and dysregulated immune response is believed to be one of the main factors behind postinfection complications.

Epidemiological data have established an association between the use of nonsteroidal anti-inflammatory drugs (NSAIDs) and CDI (11). Muñoz-Miralles and colleagues demonstrated that the NSAID indomethacin (Indo) significantly increased the severity of CDI in antibiotic-treated mice when the NSAID was applied following inoculation and throughout the infection (12), and indomethacin exposure is associated with alterations in the structure of the intestinal microbiota (13, 14). NSAIDs are among the most highly prescribed and most widely consumed drugs in the United States (15), particularly among older adults (16), and have been implicated in causing spontaneous colitis in humans (17, 18). They act by inhibiting cyclooxygenase (COX) enzymatic activity, which prevents the generation of prostaglandins (PGs) and alters the outcome of subsequent inflammatory events. Prostaglandins, especially PGE_2_, are important lipid mediators that are highly abundant at sites of inflammation and infection and that support gastrointestinal homeostasis and epithelial cell (EC) health (19). NSAID use has been associated with shifts in the gut microbiota, in both rodents and humans (20–23), but these shifts have not been explored in the context of CDI.

In this report, we deployed a mouse model of antibiotic-associated CDI to examine the impact of exposure to indomethacin prior to infection with C. difficile on disease severity, immune response, intestinal epithelial integrity, and the gut microbiota. These investigations revealed that even a brief exposure to an NSAID prior to C. difficile inoculation dramatically increased CDI severity, reduced survival, and increased pathological evidence of disease. Inhibition of PG biosynthesis by indomethacin altered the cytokine response and immune cell recruitment following CDI, enhancing intestinal tissue histopathology and allowing partial systemic bacterial dissemination by dismantling intestinal epithelial tight junctions (TJs). Additionally, indomethacin treatment alone significantly perturbed the structure of the gut microbiota. These findings support epidemiological data linking NSAID use and CDI and caution against the overuse of NSAIDs in patients at high risk for C. difficile, such as older adults.

## RESULTS

### Indomethacin worsens C. difficile Infection in Mice and Increases Mortality.

**FIG 1:**
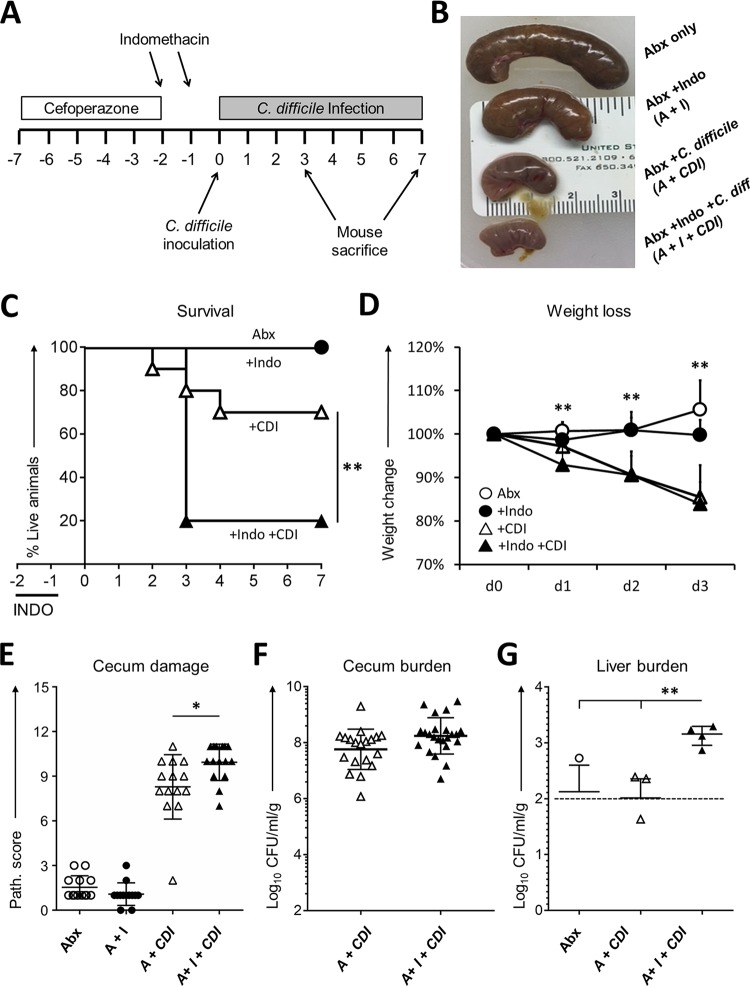
Indomethacin worsens the effects of C. difficile infection in mice. (A) C57BL/6 mice were treated with cefoperazone for 5 days followed by 2 days of recovery and then challenged by gavage with 1 × 10^4^ spores of NAP1 strain M7404. Animals received 2 doses of 10 mg/kg of body weight of indomethacin by gavage daily as indicated by the top arrows. (B) Representative picture illustrating the macroscopic effects of the different treatments in the cecum. Indo, indomethacin; Abx, antibiotic; *C. diff*, C. difficile. (C to E) Mice were monitored for survival (Kaplan-Meier curve) (C), weight loss (D), and histopathologic severity of colitis (E) (*n* = 13 to 15/group). (F and G) C. difficile bacterial burden was evaluated in the ceca of 12 mice/group (F) and total aerobic bacterial burden plus anaerobic bacterial burden in the liver of 5 mice/group (G) also at day 3 after infection, with the discontinuous line indicating the limit of detection. Path., pathology. **, P < 0.01 (by log rank [Mantel-Cox] test for survival [panel C] and by unpaired *t* test for weights [panel D]); *, P < 0.05 (1-way analysis of variance [ANOVA] test for histopathological scores [panel E]); **, P < 0.01 (Wilcoxon test with Bonferroni correction [panel G]). I, indomethacin; A, antibiotic.

To determine the extent to which preexposure to NSAIDs influences the natural course of CDI, mice were treated with indomethacin for 2 days prior to inoculation with C. difficile (Fig. 1A). We infected C57BL/6 female mice with 1 × 10^4^ spores of C. difficile NAP1/BI/027 strain M7404 following 5 days of pretreatment with a broad-spectrum antibiotic, cefoperazone (Fig. 1A). This brief indomethacin treatment prior to CDI dramatically decreased cecum size and increased the mortality rate from 20% to 80% (Fig. 1C) but did not significantly impact weight loss (Fig. 1D). Mice pretreated with indomethacin and infected with C. difficile also displayed histopathological evidence of more-severe cecal tissue damage compared to mice infected with C. difficile that were not exposed to the drug (Fig. 1E). Indomethacin-exposed and infected mice exhibited no change in the burden of C. difficile in the cecum (Fig. 1F), but their livers harbored significantly greater loads of mixed aerobic and anaerobic bacteria (Fig. 1G), suggesting that indomethacin pretreatment compromised intestinal barrier function during CDI and fostered microbiota translocation to the liver.

### Indomethacin alters the proportions of neutrophils and CD4^+^ T cells in mucosal-associated tissues during C. difficile infection.

**FIG 2:**
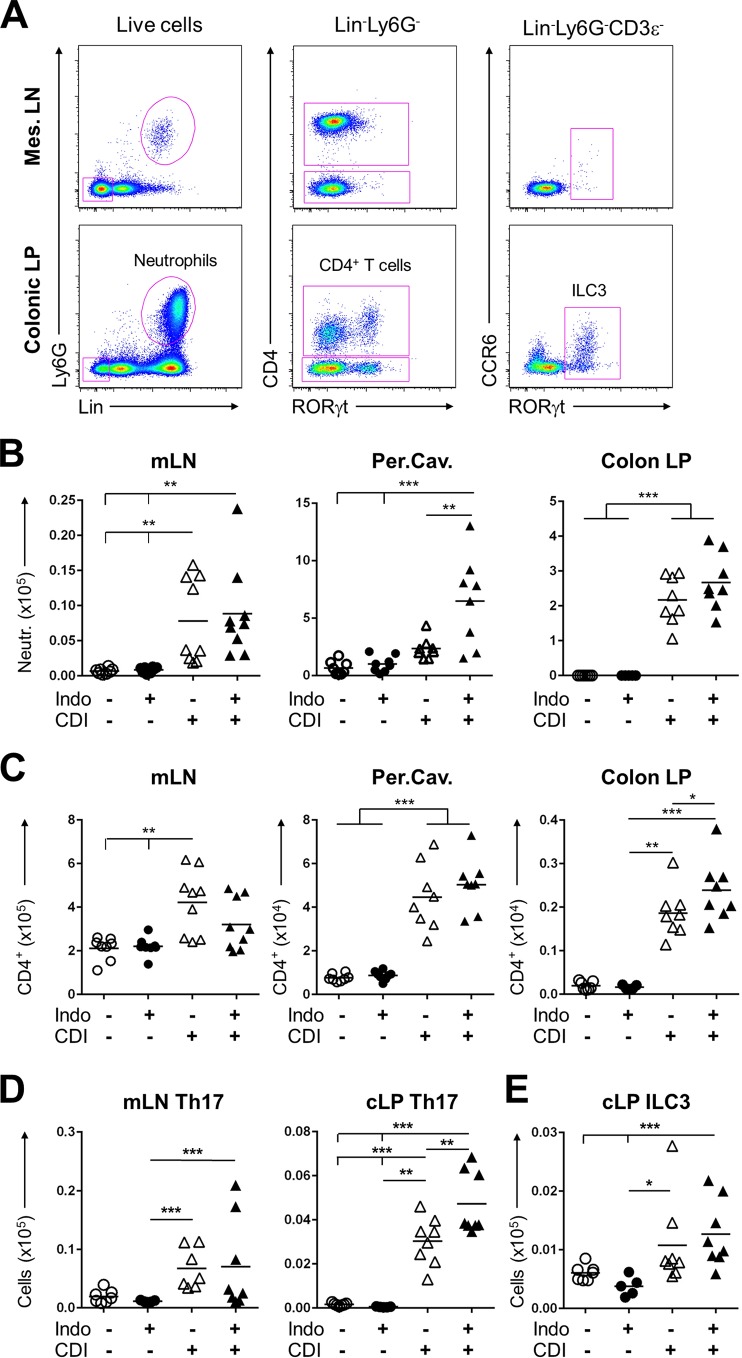
Indomethacin alters the proportions of neutrophils and CD4^+^ T cells in mucosa-associated tissues during CDI. Mice were treated as previously described and were euthanized 3 days after infection. The colon lamina propria (Colon LP), mesenteric lymph nodes (mLN), and peritoneal cavity (Per.Cav.) were collected for analysis by flow cytometry (*n* = 8 to 10/group). (A) Representative flow plots from day 3 (d3) CDI mice subjected to infection but not treated with indomethacin, depicting the gating used to identify neutrophils (Lin^+^ Ly6G^+^), CD4^+^ T cells (Lin^−^ CD4^+^), and ILC group 3 cells (Lin^−^ Ly6G^−^ CD4^−^ RORγt^+^) in different organs. Colonic LP, colon lamina propria; Mes. LN, mesenteric lymph nodes. (B and C) Neutrophil (Neurtr.) numbers (×10^6^) (B) and CD4^+^ T cell numbers (×10^6^) (C). (D and E) Quantification of results of the analysis of mLN and cLP CD4^+^ RORγt^+^ Th17 cells (D) and ILC type 3 (Lin^−^ CD3ε^−^ RORγt^+^) cells (E). The horizontal middle line in each column represents the average. One-way analysis of variance (ANOVA) with Turkey’s correction was used to evaluate significant differences among all groups. *, P < 0.05; **, P < 0.01; ***, P < 0.001.

The mucosal immune response is an important factor in the clearance of and the pathology associated with CDI (10, 24–27). NSAIDs can disturb immune homeostasis within the gastrointestinal mucosa (28) and have been used to trigger immune-mediated colitis in mice(29). We determined the extent to which indomethacin altered immune cell populations in and around the gastrointestinal tract during CDI. Mice were euthanized by day 3 after infection, and cells from the peritoneal cavity, mesenteric lymph nodes (mLN), and colonic lamina propria (cLP) were processed for flow cytometry analysis. CDI provoked an increase of neutrophil and CD4^+^ T cell numbers across all three compartments (Fig. 2). Focusing on the differences caused by indomethacin exposure prior to CDI, we found that neutrophils levels were significantly increased in the peritoneal cavity compared to those seen with CDI alone. This was paralleled by a similar overall trend in the mLN and colonic lamina propria (Fig. 2B). On the other hand, numbers CD4^+^ T cells were slightly decreased in the mLN but were increased in the cLP (Fig. 2C), possibly due to selective migration and/or proliferation in inflamed sites. Considering that IL-17 has been implicated in driving the neutrophilic inflammatory response to CDI (88) and that Th17 cells and innate lymphoid cells, type 3 (ILC3), are major sources of IL-17 during inflammatory responses, we evaluated the combined effects of indomethacin and CDI on these populations. Interestingly, higher numbers of CD4^+^ RORγt^+^ (Th17) cells were found in the cLP but not in the mLN (Fig. 2D). CDI also induced a modest expansion of ILC3 cell numbers in the cLP, but there were no significant alterations due to indomethacin pretreatment (Fig. 2E). These data demonstrate that indomethacin pretreatment exacerbates neutrophilic and Th17-type immune responses in the intestinal milieu to CDI in the mouse.

### Indomethacin dysregulates the expression of genes involved in prostaglandin metabolism and inflammatory peptides during CDI.

**FIG 3:**
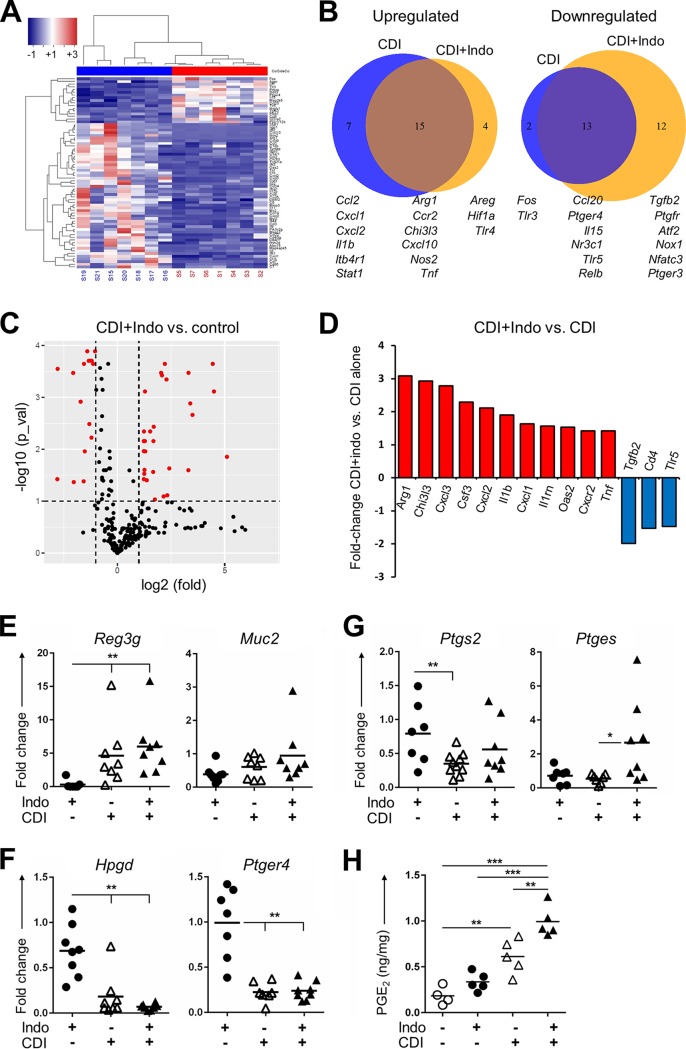
Prostaglandin inhibition by indomethacin inhibits an intestinal protective PGE_2_-mediated response to Clostridium difficile and induces damage driven by innate immune cells. (A) Representative clustering showing relative mRNA expression levels comparing (*x* axis) the groups that received CDI alone (red) to those that received control cefoperazone only (blue) from one experiment performed with *n* = 7/group at day 3 of treatment. (B) Venn diagram depicting overlap in gene upregulation and downregulation upon CDI or CDI^+^ indomethacin pretreatment compared to control mice. The size of each circle is proportional to number of genes. (C) Volcano plot of CDI^+^ indomethacin treatment results versus control results. Red dots in the volcano plots represent significantly differently expressed genes that were either underexpressed or overexpressed. The horizontal line represents the adjusted *P* value cutoff of 0.1; the vertical lines represent fold cutoff values of 0.5 and 2. The red dots represent the winner genes. (D) Summary of gene data, depicting the highest fold differences in upregulation (red) and downregulation (blue) in comparisons of the CDI^+^ indomethacin group to the CDI-alone group. Data from panels B to D were pooled from 2 experiments with *n* = 11 to 12 samples/group. (E to G) Relative mRNA expression levels of intestinal markers of inflammation and protection *Reg3g* and *Muc2* (E), the degrading PGE_2_ catabolic enzyme 15-PGHD (*Hpgd*) and PGE_2_ receptor EP4 *(ptger4*) (F), and the inducible enzymes controlling PGE_2_ metabolism COX2 and mPGES (*Ptgs2* and *Ptges*) (G). Ceca from mice undergoing treatment were used to obtain mRNA, generate cDNA, and perform RT-PCR at day 3 post-CDI from 8 mice/group pooled from 2 experiments.*, P < 0.05; **, P < 0.01 (1-way ANOVA). (H) PGE_2_ concentration (in nanograms per milligram of tissue) in the supernatants of colon explants cultured for 12 h from the mice in the indicated treated groups (*n* = 4 to 5/group). **, P < 0.05; ***, P < 0.01 (heteroscedastic unpaired *t* test).

CDI induces extensive transcriptional changes in the intestines that generally result in protective responses that restrain bacterial spread and mitigate induced intestinal epithelial pathology (3, 30). To examine the impact of indomethacin on this response, we interrogated transcriptional changes related to inflammatory responses in the cecum following indomethacin pretreatment followed by CDI using a panel of inflammatory markers (NanoString nCounter) that encompassed 254 transcripts. After curating the processed raw data to remove false positives/negatives, to normalize the data, and to filter for internal coherence with 6 housekeeping genes, we obtained mRNA values for 168 genes and generated a list of genes significantly altered upon exposure to each treatment compared to the antibiotic-treatment-alone group. We noted significant alterations, both positive and negative, in the inflammatory gene transcriptome of the cecum in mice infected with C. difficile following brief indomethacin exposure compared with C. difficile-inoculated mice that were not treated with the NSAID (Fig. 3B to D). Notably, indomethacin pretreatment followed by CDI significantly upregulated several genes involved in innate immune cell activation and recruitment such as *Il1b*, *Cxcl3, Csf3*, and *Cxcl1* and downregulated *Cd4*, *Tlr5*, and *Tgfb2* (Fig. 3C and D).

To further characterize the impact of NSAIDs on the immune response during CDI, we explored the impact of indomethacin on intrinsic mechanisms of host defense in the gastrointestinal tract. Specifically, we focused on the Gram-positive selective antimicrobial peptide REG3γ and on mucin, two host intestinal defense factors that have been shown to be important for the control of gastrointestinal infections (31). We confirmed by quantitative reverse transcription-PCR (qRT-PCR) that CDI upregulated *Reg3g* transcription whereas *Muc2* transcript levels were not significantly altered following indomethacin treatment (Fig. 3E). To evaluate if PGE_2_ synthesis and signaling were altered due to infection or indomethacin treatment, we analyzed the expression of genes encoding PGE_2_ receptors and the enzymes involved in PGE_2_ metabolism. The transcription of PGE_2_ receptor gene *Ptger4* was severely suppressed upon CDI, but indomethacin did not significantly exacerbate the suppression (Fig. 3F). Infection with C. difficile suppressed colonic expression of the *Ptgs1* and *Ptgs2* genes, encoding COX-1 and COX-2, respectively (Fig. 3G). Notably, indomethacin pretreatment prevented this downmodulation and simultaneously induced the expression of the *Ptges* gene, which encodes an inducible synthase for PGE_2_ (Fig. 3G). What is more, indomethacin further reduced expression of the gene encoding PGE_2_-inactivating enzyme 15-hydroxyprostaglandin dehydrogenase (*Hpgd* gene; Fig. 3G). This selective inhibition of *Ptgs2* transcription, together with inhibition of *Hpgd* and enhancement of *Ptges*, is consistent with the paradoxical increase in PGE_2_ concentrations that we observed at 72 h after infection in the supernatant of colon explants from mice treated in the same manner (Fig. 3H). Together, these data demonstrate that indomethacin pretreatment increases innate immune cell activation and recruitment while also leading to PG dysregulation.

### Indomethacin increases intestinal inflammation by upregulating a combination of myeloid cell recruitment and response in the cecum.

**FIG 4:**
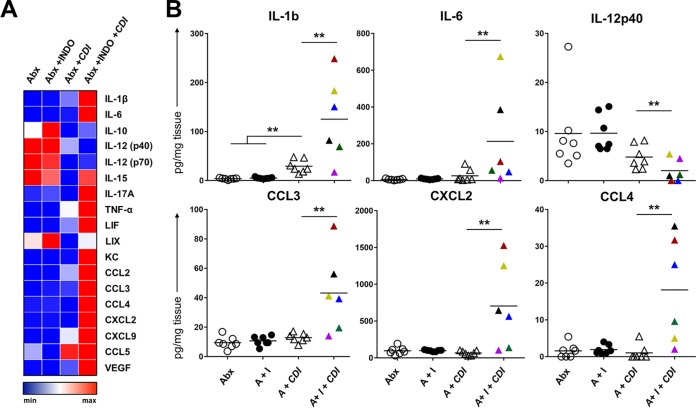
Indomethacin treatment enhances the inflammatory milieu in ceca of mice infected with C. difficile. Mice were treated as described for Fig. 3 and 4 and their ceca collected at day 3 after infection. (A) Protein expression levels in homogenates from individual ceca were measured by Luminex assay and plotted on a log-2 scale. *n* =7 to 8/group. All values are provided in picograms of protein/cecum protein content. VEGF, vascular endothelial growth factor receptor. (B) Selected proinflammatory cytokines and myeloid cell-recruiting chemokines plotted to depict the range of variation. Filled triangles in the A ^+^ I^+^ CDI group are color-coded to match individual samples.

Following the observation that indomethacin pretreatment significantly altered cellular and transcriptional immune responses during CDI, we sought to determine the impact of this drug on tissue-level inflammatory protein expression during infection. Infected mice (exposed or not exposed to indomethacin) were euthanized, and ceca were harvested at day 3 post-CDI. Whole-tissue homogenates were used to measure the concentration of a panel of inflammation-related proteins and were normalized to total protein content per cecum (Fig. 4). The protein levels observed largely supported the transcriptomics results from our previous studies, confirming what has already been reported for CDI regarding IL-1β and immune mononuclear cell recruitment and activation proteins such as CCL3, CXCL2, and CCL4. Interestingly, class IL-6 cytokines (IL-6, leukemia inhibitory factor [LIF]) were among those whose numbers were most enhanced by indomethacin pretreatment, consistent with what has been found in humans infected with C. difficile (25, 32, 33). Together with the increase in IL-1β, and consistent with the results reported above showing enhanced Th17 responses, these data imply that an exacerbated IL-17A-related response caused by indomethacin had occurred. In contrast, some type-1-associated inflammatory molecules such as IL-12p40 were downregulated by indomethacin pretreatment.

### Indomethacin perturbs colonic epithelial cell junctions of C. difficile-infected mice.

**FIG 5:**
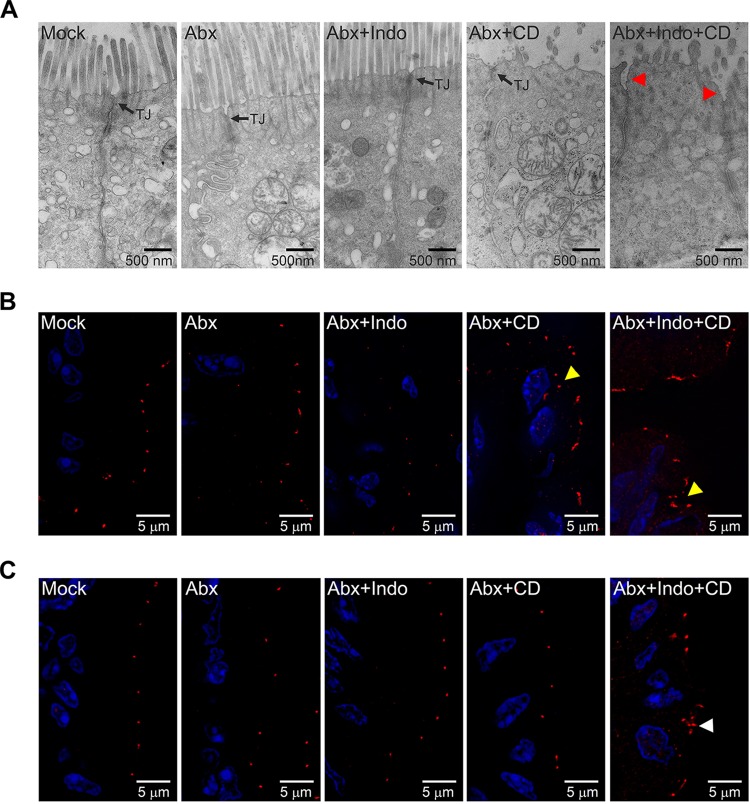
Indomethacin promotes relocalization of TJ-associated protein ZO1 and perturbs colonic epithelial cell junctions of C. difficile-infected mice. (A) Transmission electron micrographs showing lateral views of colonic mucosa from untreated control mice (Mock) or mice treated with the following: cefoperazone alone (Abx); cefoperazone and indomethacin (Abx + Indo); cefoperazone and C. difficile (Abx + CD); or cefoperazone, indomethacin, and C. difficile (Abx + Indo + CD). Arrows point to intact tight junctions (TJ). Red arrowheads point to TJ unzipping or separation. (B and C) Mouse colonic tissues from the groups described above were stained for TJ-protein occludin (B) and TJ-associated protein ZO1 (C). Occludin and ZO1 stain are pseudocolored in red. DAPI (blue) was used to stain DNA. Yellow arrowheads and white arrowheads indicate cytoplasmic relocalization of occludin and ZO1, respectively (see also Fig. S1).

The observations of the increased bacterial translocation (Fig. 1G), together with the increased local PGE_2_ levels (Fig. 3H) and enhancement of numbers of inflammatory molecules (Fig. 4), led us to investigate whether the integrity of the intestinal epithelial barrier was compromised due to indomethacin pretreatment during CDI. We assessed the impact of indomethacin on the integrity of colonic epithelial junctions of C. difficile-infected mice via transmission electron microscopy and immunofluorescence staining of tight junction (TJ) proteins and TJ-associated proteins. Intestinal epithelial cells (IECs) of uninfected mice, cefoperazone-treated and uninfected mice, and cefoperazone-and-indomethacin-pretreated mice had uniform microvilli and intact cell junctions similar to those seen with mock-treated mice (Fig. 5A). C. difficile infection resulted in effacement of microvilli of intestinal epithelial cells but did not appear to cause gross structural alterations of the cell junctions. In contrast, indomethacin pretreatment of C. difficile*-*infected mice triggered striking intestinal epithelial cell separation at the region of the TJs.

10.1128/mBio.02282-18.1 FIG S1 Occludin and ZO-1 superposition with DAPI and bright-field microscopy. Mice were treated as described above, and microscopy images were acquired as described for Fig. 5, with DAPI results shown in blue and occludin or ZO-1 results in red. The corresponding superimposition is shown here with bright-field microscopy to delineate intestinal epithelial borders and to enable appreciation of structural details. Download FIG S1, TIF file, 3.4 MB. Copyright © 2019 Maseda et al. 2019 Maseda et al. This content is distributed under the terms of the Creative Commons Attribution 4.0 International license.

TJ complexes containing membrane-anchored occludin, claudins, and junctional adhesion molecules (JAMs) attach to the perijunctional actomyosin ring via adaptor proteins such as zona occludens 1 (ZO1). Consistent with the intact cell junctions observed in IECs of uninfected mice, occludin and ZO1 localized at the apex of lateral cell junctions (Fig. 5B and C). In contrast, CDI resulted in occludin relocalization to the cytoplasm of epithelial cells. ZO1 redistribution to the cytoplasm, however, was observed only in C. difficile-infected mice that were previously treated with indomethacin (see Fig. S1 in the supplemental material). Collectively, our data suggest that indomethacin acts synergistically with C. difficile to alter the localization of occludin and ZO1 and to perturb the TJ integrity of intestinal epithelial cells *in vivo*.

### Indomethacin alters the intestinal microbiota composition without further reducing microbial community diversity after antibiotic treatment.

**FIG 6:**
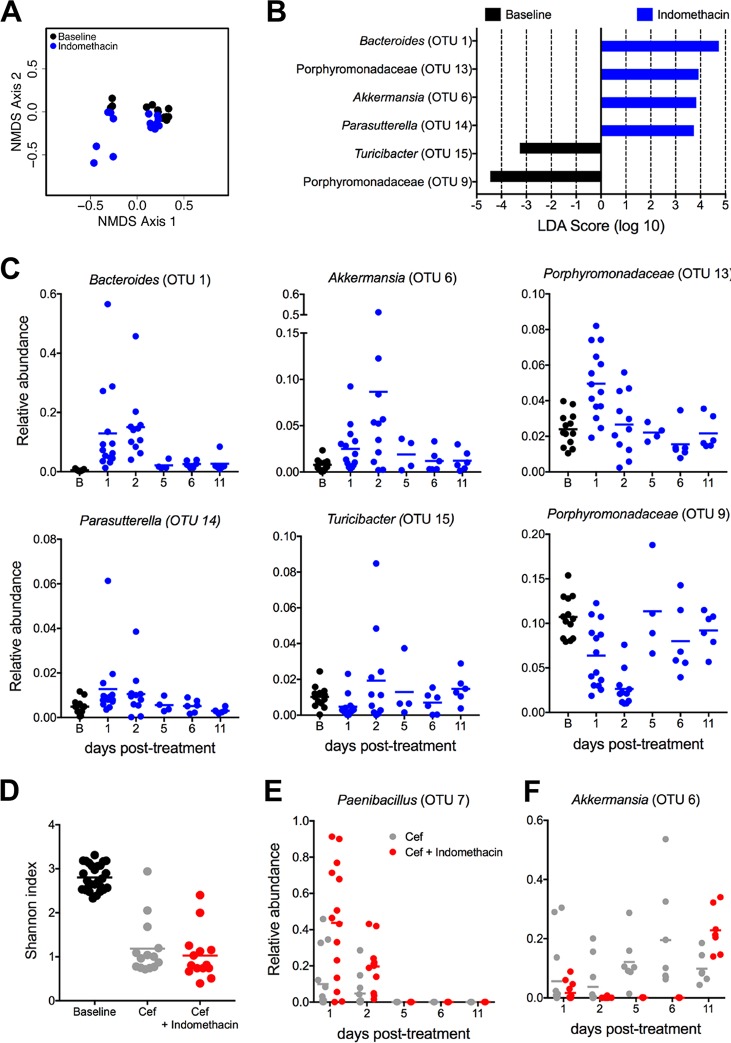
Indomethacin treatment alters the gut microbiota. (A) Nonmetric multidimensional scaling (NMDS) ordination showing β-diversity as measured by Yue and Clayton’s measure of dissimilarity (*θ*_YC_) on day 1 post-indomethacin treatment. The significance of results of comparisons between baseline (black) and indomethacin-treated (blue) samples was determined using analysis of molecular variance (AMOVA) (*P* < 0.001). (B) Differentially abundant taxa in baseline and indomethacin-treated animals ranked by effect size. (C) Dynamics of recovery of differentially abundant taxa over an 11-day time course. Sample sizes: column B (baseline microbiota pretreatment), *n* = 13; column d1, *n* = 14; column d2, *n* = 11; column d5, *n* = 4; column d6, *n* = 6; column d11, *n* = 6. (D) Shannon diversity index for untreated (baseline; black), cefoperazone-treated (gray), or cefoperazone-and-indomethacin-treated (red) mice (see also Fig. S2). (E and F) Dynamics of *Paenibacillus* (E) and *Akkermansia* (F) relative abundances following cefoperazone treatment (gray) and cefoperazone-plus-indomethacin treatment (red) (see also Fig. S3).

The composition of the gut microbiota has a profound impact on the manifestation and clearance of CDI, as well as on the virulence of C. difficile and on the outcome of disease (34, 35). There is also evidence suggesting prominent off-target effects of pharmaceutical agents, such as NSAIDs, on the gut microbiota and on gastrointestinal health (21, 36). To examine the impact of indomethacin on the murine gut microbiota, mice were treated with a 2-day course of indomethacin and the microbial community was subsequently surveyed using 16S rRNA gene sequencing. At 1 day posttreatment, mice given indomethacin showed no significant alteration in α-diversity (Fig. S2) but exhibited a significant shift in community structure compared to untreated mice (*P* < 0.001 [analysis of molecular variance {AMOVA}]) (Fig. 6A). To characterize differentially abundant taxa in indomethacin-treated mice, we utilized the biomarker discovery algorithm LEfSe (linear discriminant analysis [LDA] effect size). Indomethacin treatment was associated with an enrichment in numbers of operational taxonomic units (OTUs) affiliated with the *Bacteroides* (OTU 1)*, Akkermansia* (OTU 4), and *Parasutterella* (OTU 17) genera and with the *Porphyromonadaceae* (OTU 14) family (Fig. 6B and C). Moreover, we observed a significant decrease in OTUs affiliated with the *Turicibacter* (OTU 18) genus and *Porphyromonadaceae* (OTU 5) family following indomethacin treatment (Fig. 6B and C). To examine the longitudinal impact of indomethacin on the murine gut microbiota, we collected samples periodically for 11 days following administration of indomethacin. We observed significant differences in community structure at up to 2 days following administration of indomethacin treatment, and a significant enrichment of *Bacteroides* (OTU 1) could be detected as much as 11 days following treatment with indomethacin (Fig. 6C). Next, to determine how indomethacin may impact the microbiota in the context of antibiotic treatment, mice were again exposed to indomethacin for 2 days following 5 days of cefoperazone (0.5 mg/ml) treatment. Although cefoperazone treatment dramatically reduced the overall level of community α-diversity in all mice, we detected significant alterations in community structure associated with cotreatment with indomethacin and cefoperazone that could be observed at 11 days posttreatment (Fig. 6D; see also Fig. S3). Initial differences in microbial community structure were driven by a significant bloom in *Paenibacillus* (OTU 11), while *Akkermansia* (OTU 6) was significantly enriched in mice treated with indomethacin and cefoperazone on day 11 posttreatment (Fig. 6E and F). Together, these data suggest that indomethacin has a marked effect on the structure of the gut microbiota and that these off-target effects likely contribute to disease exacerbation during CDI.

10.1128/mBio.02282-18.2 FIG S2 Indomethacin does not alter the microbiota α-diversity. Mice were treated with indomethacin for 2 days and stool samples collected for microbiota analysis. α-Diversity levels were measured by Shannon diversity index analysis for untreated mice (baseline; black) and indomethacin-treated mice (blue). Download FIG S2, TIF file, 0.1 MB. Copyright © 2019 Maseda et al. 2019 Maseda et al. This content is distributed under the terms of the Creative Commons Attribution 4.0 International license.

10.1128/mBio.02282-18.3 FIG S3 Indomethacin alters the gut microbiota. Nonmetric multidimensional scaling (NMDS) ordinations showing β-diversity were determined by the use of Yue and Clayton’s measure of dissimilarity (*θ*_YC_) on the days after treatment indicated at the top. (A) Time course following treatment with indomethacin (*n* = 6). (B) Time course following treatment with cefoperazone (*n* = 6) or cefoperazone and indomethacin (*n* = 6). Download FIG S3, TIF file, 0.3 MB. Copyright © 2019 Maseda et al. 2019 Maseda et al. This content is distributed under the terms of the Creative Commons Attribution 4.0 International license.

## DISCUSSION

CDI is the most commonly diagnosed cause of antibiotic-associated diarrhea and has surpassed methicillin-resistant Staphylococcus aureus as the most common health care associated infection in many U.S. hospitals (37). Nearly 30,000 people die each year in the United States from CDI (38). A major challenge of CDI is recurrence, which can impact 20% to 30% of patients and is associated with an increased risk of death (4, 39). One of the most promising treatments for CDI is fecal microbiota transplantation (FMT), which is estimated to be >80% effective in most studies (40, 41). However, problems with standardization and availability and putative risks from FMT have made this form of therapy suboptimal (42). There continues to be a demand for effective approaches to limit CDI severity and to understand complications arising from the synergy of CDI with intestinal immune responses and with the drugs used to limit damaging inflammatory effects.

The NSAIDs are among the most commonly prescribed drugs in the United States, with more than 98 million prescriptions filled annually (15), and an estimated 29 million Americans use over-the-counter NSAIDs per year (43). As they prevent synthesis of endogenous PGs, NSAIDs can adversely affect intestinal health. Results from epidemiological studies, underscored by a recent meta-analysis (11), have revealed an association between CDI risk and the use of NSAIDs. The plausibility of a link between NSAID use and CDI is bolstered by the association between NSAID use and flare-ups of inflammatory bowel disease and the occasional occurrence of NSAID-induced colitis (44–47). Recent mouse studies have established that concomitant NSAID use exacerbates active CDI (12).

Animal and human studies suggest that CDI induces local and systemic increases in PGs, including PGE_2_ (48). Prostaglandin E_2_ is one of the most common and best-characterized PGs and has long been known to have major effects on gastrointestinal health (49–52). COX-1-dependent production of PGE_2_ is gastroprotective, explaining why chronic NSAID use is associated with stomach ulcers and why such ulcers can be prevented by administering the FDA-approved oral PGE analogue misoprostol to NSAID-treated patients (53). In addition, endogenous PGE_2_ production prevents gut epithelial cell death and promotes colonic tumor growth by directly inducing tumor epithelial cell proliferation, survival, and migration invasion (53–55). It is also possible that PGE_2_ modulates disease through alteration of the microbiome, as NSAIDs have been implicated as potentially disrupting the gut microbiome (21, 56). Additionally, PGE_2_ functions as a key inflammatory signal that can regulate certain immune responses, with its local levels being tightly regulated during the trigger of but also the resolution of inflammatory processes (57–59). Some of the best-known functions of PGE_2_ are indeed its role in intestinal inflammation and cancer and its impact on the immune system (19). Paradoxically, we observed that pretreatment with the COX inhibitor indomethacin caused a dysregulation of PG metabolism that led to increased PGE_2_ production upon CDI. This heightened PGE_2_ production might represent a “rebound” effect following temporary exposure to indomethacin prior to infection, since continued indomethacin dosing throughout infection was recently shown to suppress PGE_2_ production (12). This exaggerated PGE_2_ response was associated with elevated levels of intestinal inflammatory cytokines, monocyte and neutrophil recruitment, partial dismantling of the intestinal epithelial cell’s tight junctions, and a specific disturbed microbiota composition. Interestingly, this is highly consistent with several studies that reported a protective role for PGE_2_ in inducing an orchestrated tolerance response that involves innate immune cells such as ILC3 cells, neutrophils, and macrophages (58, 60, 61).

Immune protection against C. difficile challenge seems function independently of the presence of CD4^+^ cells, anti-toxin IgG, and pIgR (62), but it strongly relies on rapid and effective myeloid cell responses (9, 38, 63). Cells of the immune system can exert critical roles in controlling bacterial pathogen damage and intestinal health through production of damaging or protective cytokines by T cells or innate lymphoid cells (ILCs) (64, 65). Production of gamma interferon (IFN-γ) by T cells and neutrophils has a role in protection against CDI (66, 67), and the associated production of IL-12 by innate cells upon CDI can have a strong positive-feedback effect on IFN-γ production in this context. The role of IL-17 cytokines and of their cellular sources is more controversial, as they can induce damage but can also trigger intestinal repair processes and maintain barrier integrity (68, 69). Perturbation of the microbiota induced by antibiotic treatment can also cause an imbalance of protective Treg/Th17 ratios (70). ILCs are, however, critical for controlling the acute response induced by CDI. In contrast to Rag1^−/−^ mice, Rag2^−/−^ Il2rγ^−/−^ mice rapidly succumb to CDI. While ILC3 cells display a limited role in resistance, loss of IFN-γ-expressing ILC1s in Rag1^−/−^ mice increased susceptibility (7). The contribution to CDI pathogenesis by other highly relevant cytokines such as IL-23 and IL-22 provided by innate immune cells strongly depends on context (24, 25, 38, 71–73). In our studies, we found that indomethacin pretreatment prior to CDI increased local levels of chemokines that induce recruitment of inflammatory myeloid cells such as CXC2, CCL3, and CCL4, with a concomitant increase in numbers of circulating and local neutrophils, while type 3 ILC numbers were unaltered. Also, in coordination with the increased levels of intestinal IL-6 and IL-1β, higher total numbers of CD4^+^ cells and CD4^+^ RORγt^+^ cells were found in the colonic lamina propria but not the draining mesenteric lymph nodes.

Intestinal epithelial cells constitute the main barrier against infectious agents colonizing the gastrointestinal tract. Cell junctional complexes, notably, the tight junctions, regulate paracellular permeability and restrict the translocation of luminal microbes and microbial products across the epithelial monolayer (74). Displacement of occludin, but not ZO-1, from the junctions of mouse colonic epithelial cells during CDI did not manifest as gross morphological changes of TJ regions during EM visualization. This is reminiscent of the alterations seen in anti-CD3-treated mice or tumor necrosis factor alpha (TNF-α)-treated mice and is consistent with the view that occludin is a regulator, rather than a key structural component, of TJs (75). However, indomethacin pretreatment with CDI redistributed both occludin and ZO-1 to the cytosol, and electron micrographs revealed a concomitant loss of TJ interactions. These changes are expected to increase paracellular permeability and promote bacterial translocation and could explain the observed increase in bacterial burden in the livers of indomethacin-treated, C. difficile*-*infected animals.

Induction of severe colitis upon CDI is subordinated to alterations in the microbiota caused by antibiotic administration that lead to dismantling of colonization resistance (5, 34, 38, 76, 77). Interestingly, recent studies have begun to highlight previously underappreciated and potentially detrimental effects of pharmaceutical drugs, such as NSAIDs, on the gut microbiota (21, 36). We observed that indomethacin did not cause an alteration of the microbial α-diversity but did induce significant alterations in microbiota structure that lead to an enrichment of *Bacteroides*, *Akkermansia*, *Porphyromonadaceae*, and *Parasutterella* populations and to a decrease in *Turicibacter* and *Porphyromonadaceae* populations. Interestingly, increases in *Bacteroides* and *Akkermansia* populations have been reported to have occurred in association with inflammatory bowel disease and other infections (78–81). Furthermore, *Turicibacter* has been shown in several studies to be associated with colonization resistance to C. difficile (6, 82). Thus, indomethacin-mediated alterations in the microbiota may have a profound impact on the manifestation and severity of CDI. Interestingly, when the microbiota α-diversity was severely reduced by antibiotic treatment, we found that *Paenibacillus* and *Akkermansia* populations expanded in mice pretreated with indomethacin. The role of *Paenibacillus* in the pathology of CDI is unknown at present, and further investigation is warranted.

Injury to intestinal epithelial barriers and microbial translocation can lead to a systemic response that mimics some aspects of sepsis and unveils a massive release of inflammatory cytokines, such as IL-1β, that increases neutrophil and macrophage recruitment and activation (25). It is still unclear how the innate and adaptive arms of the immune response coordinate during CDI, especially in situations such as the one we present with pretreatment with indomethacin. In such circumstances, it is important that type 3 ILCs can dysregulate adaptive immune CD4^+^ cell responses against commensal bacteria but that this ILC-mediated regulation of adaptive immune cells occurs independently of IL-17A, IL-22, or IL-23 but is dependent on antigen presentation (83). Additionally, administration of antibiotics alone can promote inflammation through goblet cell-mediated translocation of native colonic microbiota in mice (84), but CDI induces significant goblet cell loss (85), which would have a countering effect on bacterial translocation under experimental conditions such as those described in this work.

Our results highlight the capacity of a short-term oral dose of the NSAID indomethacin to cause an imbalance in PG production and to disrupt the intestinal barrier to allow bacterial entrance in the bloodstream. These effects are paralleled by a specific disarrangement of the intestinal microbiota and by dysregulated inflammatory and immune responses that lead to increase pathological damage and finally unfold in an increased mortality rate. Our results call for caution in the use of NSAIDs in the context of C. difficile infections but also potentially when other intestinal pathogens or insults co-occur with acute inflammatory events that affect PG balances. Moreover, we also highlight how a temporary modification of a set of key inflammatory mediators such as PGs in the host can lead to significant perturbations to the resident gut microbiota. We believe that this unique combination of effects caused by indomethacin and CDI in the host and their microbiota could represent a generalized mechanism that leads to increased intestinal damage and complications when NSAIDs, or other drugs that alter key inflammatory molecules with pleiotropic effects, are used. Given the common application of NSAIDs to treat diverse inflammatory conditions, together with the disturbances in microbiota diversity and the rates of increased incidence of CDI in developed countries, the results of these studies warrant future follow-up studies in humans.

## MATERIALS AND METHODS

### Experimental animals and infection model.

All mice used in this study were obtained from Jackson Laboratories and were C57BL/6J females that were 6 weeks of age at arrival. Mice were given 2 weeks to adapt to the new facilities and avoid stress-associated responses and to allow adaptation to the in-house conditions. Mice were given cefoperazone at 0.5 mg/ml in drinking water *ad libitum* for 5 days prior to treatment with indomethacin (Cayman Chemical) at 10 mg/kg of body weight or with vehicle (phosphate-buffered saline [PBS]) for 2 consecutive days by oral gavage and then infected with 1 × 10^4^ spores of Clostridium difficile (NAP1/BI/027 strain M7404; 89) resuspended in PBS by oral gavage or were left noninfected. Noninfected mice received only cefoperazone and afterward only vehicle by oral gavage at the same time points. For some experiments, untreated mice were used to obtain unaltered cecal microbiota.

### mRNA isolation, expression analysis and qRT-PCR.

After bulk RNA was isolated from tissues with TRIzol (Life Technologies) following the manufacturer’s instructions, a Qiagen RNeasy Plus minikit was used to further purify mRNA for downstream analysis. mRNA expression was evaluated either by the use of nSolver inflammation panel mouse v2 from NanoString (https://www.nanostring.com/application/files/9414/9556/0025/LBL-10402-01_nCounter_Mouse_Inflammation_V2_Panel_Gene_List.xlsx) directly on mRNA samples or by qRT-PCR performed using an Applied Biosystems TaqMan amplification system after cDNA generation using a SuperScript VILO cDNA synthesis kit (Invitrogen). Data generated by NanoString technologies for mRNA quantification (the full gene list is available at https://www.nanostring.com/download_file/view/409/3778) were analyzed after generating raw counts that were produced using R version 3.3.2 (https://www.R-project.org/) for Fig. 3A to D. The count data produced by the n-Counter Digital Analyzer for each of the two experiments were normalized using positive controls (geometric mean method) and housekeeping genes (geometric mean method). The two sets of data were then normalized together to be combined and analyzed together. After exclusion of one Indo-plus-CDI sample (because that mouse was confirmed not to have been infected with CDI by cecal content culture), the final normalized data set included 11 control samples and 11 Indo-treated, 12 CDI-treated, and 11 CDI-plus-Indo-treated samples. A gene was included in the analyses if its counts were above the maximum count seen with the negative control in at least 5 samples in any group. Of 248 genes, 161 were included in the subsequent analysis. For each included gene, a linear regression model was fitted with “group” as the independent variable of interest. Robust standard errors were estimated using the Huber-White method for the coefficients. The mean of the expression levels determined for each group was used to calculate the fold change between groups. *P* values for testing the mean difference between groups were adjusted using the Benjamini & Hochberg method. Volcano plots were generated using the fold change values and adjusted *P* values. A gene was selected as a “winner” gene if the adjusted *P* value was lower than 0.1 and the fold change value was higher than 2 or lower than 0.5. A heat map of the winner genes was produced to visualize the clustering of samples and genes. qRT-PCRs and data quantification and analysis were performed using an Applied Biosystems 7300 Real-Time PCR system and the following TaqMan primers: Reg3g (Mm00441127_m1), Muc2 (Mm01276696_m1), Ptger2 (Mm004360516_m1), Ptger4 (Mm004360513_m1), Ptgs1 (Mm00477214_m1), Ptgs2 (Mm00478374_m1), Ptges (Mm00452105_m1), Hpgd (Mm00515121_m1), and Gapdh (Mm99999915_g1).

### Bacterial burden in mouse organs.

Liver was collected from the mouse with sanitized instruments and immediately placed in 1 ml of PBS in a 12-well plate. After the tissue was minced with scissors, 20 ml of the supernatant was drawn off and serially diluted. Dilutions were plated on sheep blood agar plates under aerobic and anaerobic conditions. After 24 h, the plates were collected and CFU levels were calculated and normalized to the weight of the liver. Cecum was also collected using sanitized instruments, and contents were expelled by applying pressure to the organ with a scalpel. The contents were then collected and put into a 1.5-ml tube. The weight of the contents was recorded; PBS was added; and the slurry was subjected to vortex mixing, serially diluted, and plated onto sheep blood agar plates (Anaerobe Systems). After 24 h, CFU counts were performed and the data were normalized to the weight of each sample.

### Tissue protein quantification, PGE_2_ measures, and multiplex analysis.

Total cecum protein was isolated from ceca prewashed with ice-cold PBS, homogenized by the use of a tissue shredder (Tissuemiser), and then centrifuged for 3 min at 8,600 × *g*. Supernatants of these preparations were submitted for Luminex analysis of the provided analytes using x-map technology via the use of a MapgPix system in combination with multiplex kits from Millipore Sigma. Total tissue protein content was quantified by detergent-compatible (DC) assay. Data were analyzed with GraphPad Prism 6.0, and heat maps were generated using Morpheus software from the Broad Institute (https://software.broadinstitute.org/morpheus/). Supernatants of colon explants were incubated for 12 h in RPMI medium supplemented with 10% fetal calf serum (FCS) and GlutaMAX in an incubator at 37°C and 5% CO_2_ saturation and were used to measure PGE_2_ concentrations with a PGE_2_ enzyme-linked immunosorbent assay (ELISA) kit (Cayman Chemical) following manufacturer’s instructions.

### Flow cytometry.

Cell suspensions from the mesenteric lymph nodes, peritoneal lavage fluid, and colon lamina propria were obtained from euthanized mice at the indicated time points. Cell suspensions were incubated with Fc-block for 15 min on ice and then surface stained with a cocktail containing monoclonal anti-mouse antibodies, including anti-CD19 (1D3), CD8a (5H10-1), anti-CD49b (DX5), anti-CD11b (M1/70), anti-CD11c (N418), and anti-CD196 (x29-2I.17) from BioLegend as well as anti-CD4 (RM4-5) and anti-Ly6G (1A8) from BD. After 30 min of incubation on ice, cells were washed, fixed, and permeabilized using a FoxP3 Fix/Perm buffer kit from eBioscience/Thermo Fisher, and intracellular staining for RORγt (clone Q31-378) was performed as a last step. Flow cytometry data were obtained with BD FACSDiva 7.0 software and .fcs 3.0 files analyzed with FlowJo software.

### Electron and immunofluorescence microscopy.

Colonic tissue samples were fixed and stored in Karnovsky’s solution (4% paraformaldehyde–PBS [pH 7.4]–1% glutaraldehyde) for at least 24 h at 4°C. Samples were neutralized with 125 mM glycine–PBS, postfixed in 1% osmium tetroxide, and sequentially dehydrated with 15%, 30%, 50%, 70%, 90%, and 100% ethanol. Samples were then infiltrated with Spurr’s resin (Electron Microscopy Sciences, Hatfield, PA). Ultrathin sections were contrasted using 2% uranyl acetate, followed by Reynold’s lead citrate, and were visualized with a FEI Tecnai Spirit transmission electron microscope (FEI, Hillsboro, OR) equipped with an Advanced Microscopy Techniques (AMT) charge-coupled-device (CCD) camera and AMT Image Capture Engine V602 software (Advanced Microscopy Techniques, Woburn, MA).

For immunofluorescence microscopy, tissue samples were frozen in OCT embedding medium (Tissue-Tek; Sakura Finetek, Torrance, CA) and stored at −80°C. OCT-mounted tissue samples were sectioned at 3 μM thickness and fixed in 4% paraformaldehyde–PBS (pH 7.4) for 20 min at room temperature. Samples were washed with PBS, permeabilized with 0.2% Triton X-100–PBS, quenched with 50 mM NH_4_Cl–PBS, and then blocked with 5% IgG-free bovine serum albumin (BSA)–PBS. Primary antibodies used were 1:50 dilutions of rabbit anti-occludin and rabbit anti-ZO1 (Abcam, Cambridge, MA). Samples were incubated with primary antisera overnight at 4°C and then washed three times with 1% IgG-free BSA–PBS. Secondary antibodies (Alexa Fluor 555-conjugated anti-rabbit IgG) were added at 8 μg/ml in 5% IgG-free BSA for 1 h. Samples were washed with PBS, stained with 4,6-diamidino-2-phenylindole (DAPI), and mounted in ProLong Diamond antifade reagent (Thermo Fisher Scientific, Waltham, MA). Images were captured using a DeltaVision Elite deconvolution microscope (GE Healthcare, Pittsburgh, PA) equipped with an Olympus 100×/1.40 oil objective, immersion oil (*n* = 1.516), and GE Healthcare Software Version 6.5.2. ImageJ 1.51j8 (National Institutes of Health, Bethesda, MA) was used to merge and pseudocolor images.

### Colon histology and pathology scoring.

Colons from experimental mice were collected at day 3 postinfection and then flushed with cold PBS, opened longitudinally, and rolled to generate Swiss rolls. The colon rolls were fixed for 5 days in 10% buffered Formalin phosphate and then transferred to 70% ethanol for 7 days. After that, the Swiss rolls were used to generate paraffin blocks that were stained with hematoxylin and eosin (H&E) and scored for the degree of injuries as described by Theriot et al. (74).

### DNA extraction, 16S rRNA gene sequencing, and gut microbiota analyses.

Fresh fecal samples were collected from individual mice prior to (baseline) and following treatment with cefoperazone, indomethacin, or a combination of cefoperazone and indomethacin. In a subset of mice (*n* = 6/group), fecal samples were collected for the time course of a posttreatment 11-day recovery period. Following collection, fecal samples were immediately put on ice and subsequently frozen for storage at −20°C. Microbial genomic DNA was extracted using a 96-well PowerSoil DNA isolation kit (Qiagen). For each sample, the V4 region of the bacterial 16S rRNA gene was amplified and sequenced using an Illumina MiSeq Sequencing platform as described elsewhere (86). Sequences were curated using the mothur software package (v1.40.3) as previously described (6, 86, 87). Briefly, the workflow that we used included generating contigs with paired-end reads, filtering low-quality sequences, aligning the resulting sequences to the SILVA 16S rRNA sequence database, and removing any chimeric sequences flagged by UCHIME. Following curation, we obtained between 9 and 83,525 sequences per sample (median = 13,161.5), with a median length of 253 bp. To minimize the impact of uneven sampling, the number of sequences in each sample was rarefied to 4,200. Sequences were clustered into OTUs based on a 3% distance cutoff calculated using the OptiClust algorithm. All sequences were classified using the Ribosomal Database Project training set (version 16), and OTUs were assigned a taxonomic classification using a naive Bayesian classifier. Significantly altered OTUs for each group were selected using the biomarker discovery algorithm LEfSe (linear discriminant analysis [LDA] effect size) in mothur (90). α-Diversity was calculated using the Shannon diversity index, and β-diversity was calculated using the *θ*_YC_ distance metric with OTU frequency data. Pairwise analysis of molecular variance (AMOVA) was used to test the statistical significance of the results of comparisons between treatment groups using the *θ*_YC_ distance metric.

### Accession number(s).

FASTQ sequence data obtained in this study have been deposited to the Sequence Read Archive (SRA) at NCBI under accession number SRP152292.
