# Supplementary figures and images for: Nonsteroidal Anti-inflammatory Drugs Alter the Microbiota and Exacerbate Clostridium difficile Colitis while Dysregulating the Inflammatory Response
Source: mBio. 2019 Jan 8;10(1):e02282-18. doi: 10.1128/mBio.02282-18 (PMC6325247; doi:10.1128/mBio.02282-18)

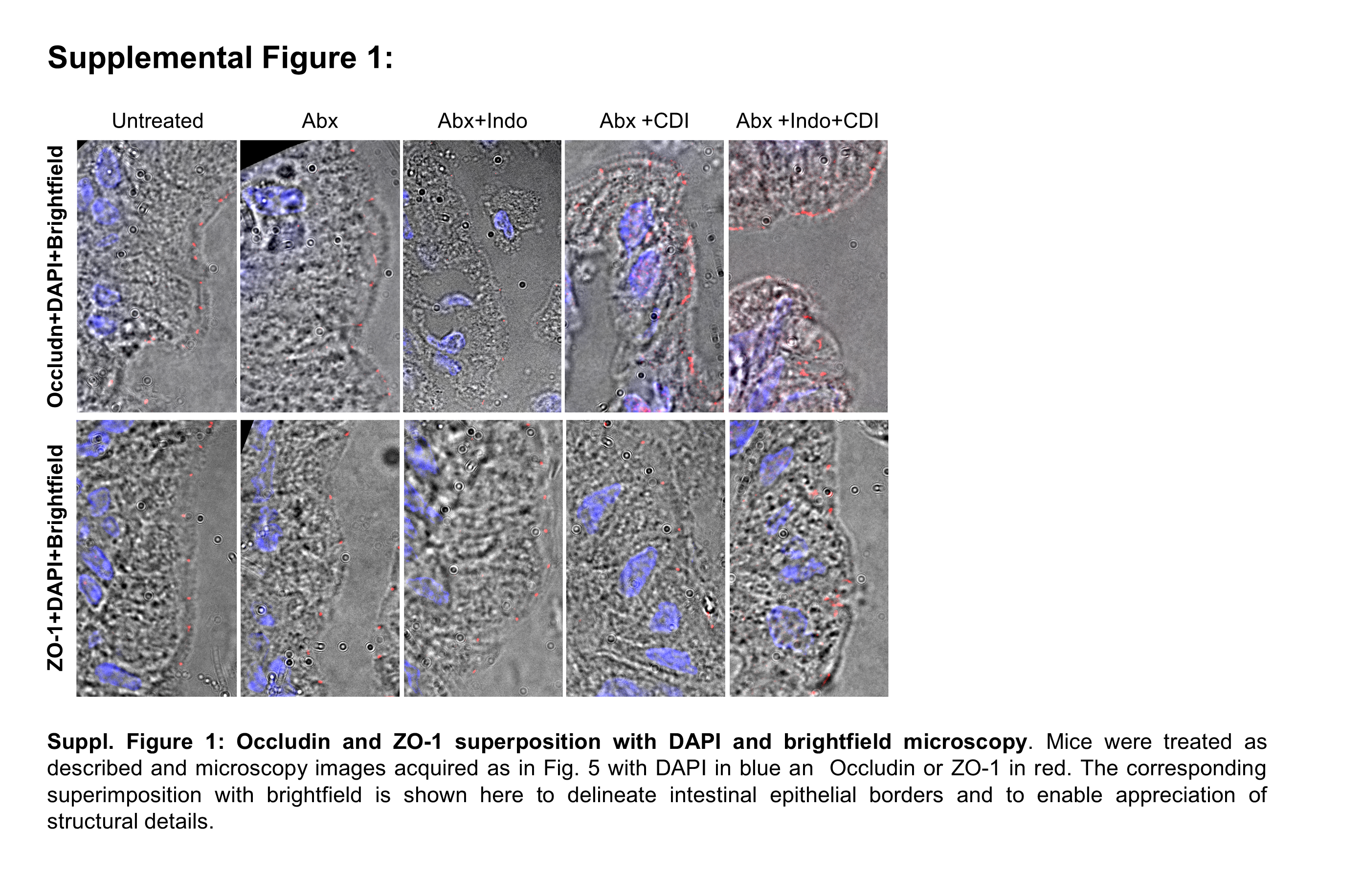

Supplement: FIG S1 [file mBio.02282-18-sf001.tif]

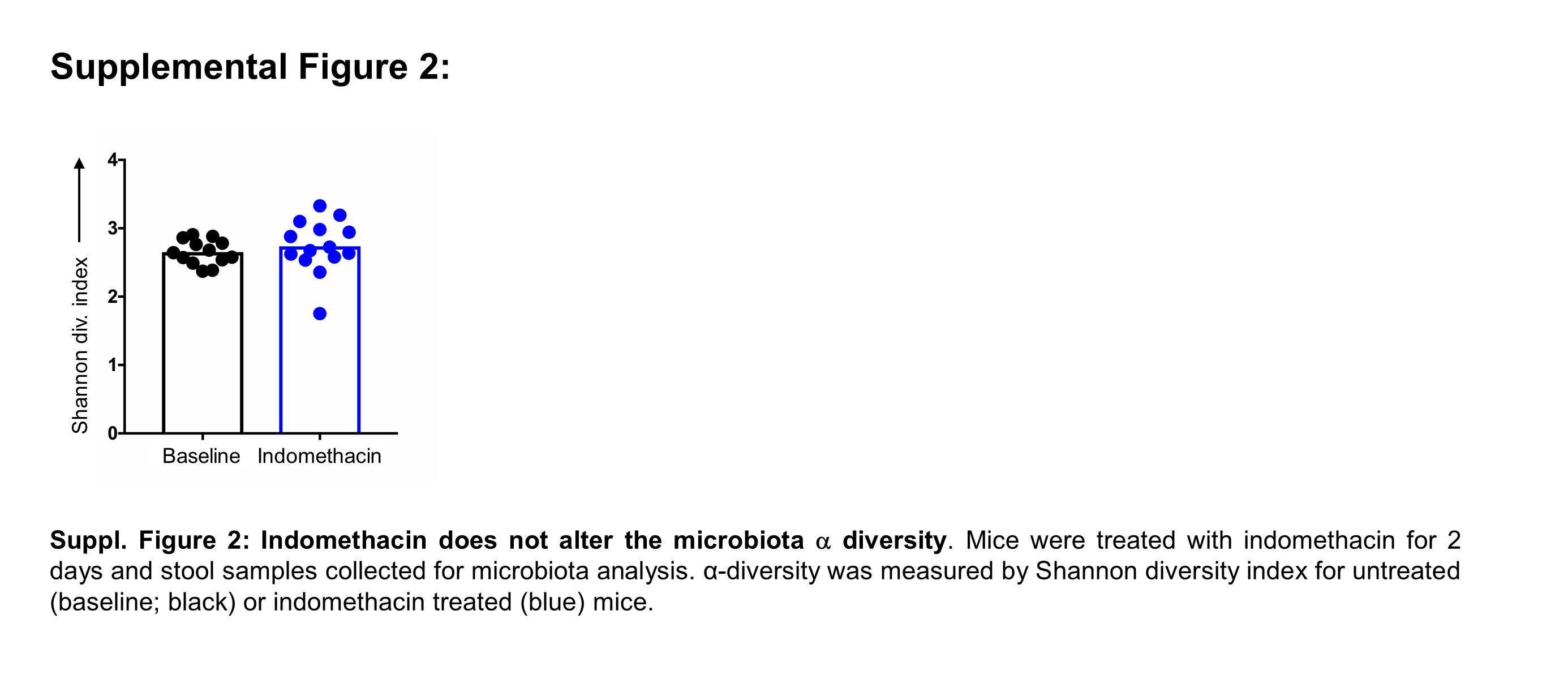

Supplement: FIG S2 [file mBio.02282-18-sf002.tif]

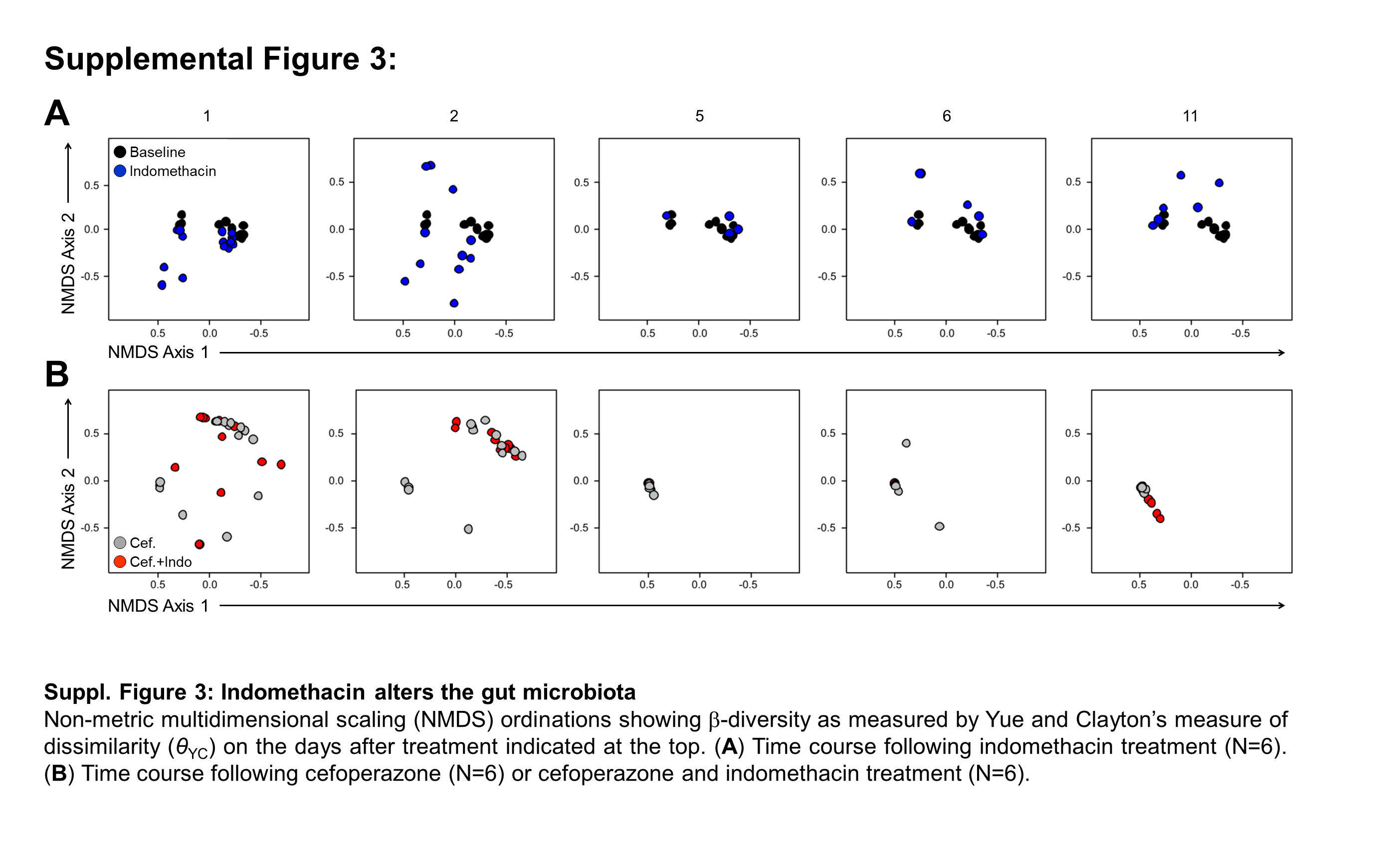

Supplement: FIG S3 [file mBio.02282-18-sf003.tif]
